# Dietary red palm oil supplementation decreases infarct size in cholesterol fed rats

**DOI:** 10.1186/1476-511X-10-103

**Published:** 2011-06-20

**Authors:** Gergo Szucs, Dirk J Bester, Krisztina Kupai, Tamas Csont, Csaba Csonka, Adriaan J Esterhuyse, Peter Ferdinandy, Jacques Van Rooyen

**Affiliations:** 1 Cardiovascular Research Group, University of Szeged, Dom ter 9, Szeged, H-6720, Hungary; 2 Department of Biomedical Sciences, Faculty of Health and Wellness Sciences, Cape Peninsula University of Technology, Symphony Way, Bellville, 7535, South Africa; 3 Pharmahungary Group, Hajnoczy u 6, Szeged, 6722, Hungary

## Abstract

**Background and Aims:**

The effect of red palm oil (RPO) supplementation on infarct size after ischaemia/reperfusion in a cholesterol enriched diet-induced hyperlipidemic animal model has not been reported. Previous studies reported results on the effect of RPO in a normal diet, whilst evidence of protection has been linked to improved functional recovery, prosurvival kinase, anti-apoptosis and NO-cGMP. Therefore, we aimed to investigate the effects of dietary RPO supplementation in a cholesterol-enriched diet-induced hyperlipidemic rat model and to investigate the involvement of matrix metalloproteinase 2 (MMP2) inhibition as a possible mechanism of protection.

**Materials and Methods:**

Male Wistar rats were fed either a standard rat chow diet (Norm) or a 2% cholesterol-enriched diet (Chol) for nine weeks. Additionally, two more groups received the same treatment, however, at the week 4, diet was supplemented with RPO for the last five weeks (Norm+RPO and Chol+RPO), respectively. After the feeding period hearts were isolated, perfused according to Langendorff and subjected to 30 minutes of normothermic global ischaemia followed by two hours of reperfusion. Infarct size was measured by 2,3,5-triphenyltetrazolium chloride staining at the end of reperfusion.

**Results:**

Cholesterol-enriched diet increased myocardial infarct size from 23.5 ± 3.0% to 37.2 ± 3.6% (p < 0.05) when compared to normal diet. RPO supplementation significantly reduced infarct size either in Norm+RPO or in Chol+RPO (to 9.2 ± 1.0% and 26.9 ± 3.0%), respectively. Infarct size in Chol+RPO was comparable to the Norm group. MMP2 activity before ischaemia was significantly reduced in the Chol+RPO group when compared to the Chol group. However, the MMP2 activity of the hearts of the RPO fed rats was significantly increased when compared to the normal diet group after ischaemia.

**Conclusions:**

For the first time it was shown that dietary RPO supplementation attenuated the increased susceptibility of the hearts in cholesterol fed rats to ischaemia/reperfusion injury. This was shown by reduced infarct size. For the first time we also show that red palm oil supplementation altered pre-ischaemic levels of MMP-2, which may indicate that myocardial MMP2 may be implicated as a possible role player in RPO mediated protection against ischaemia/reperfusion injury in hearts of cholesterol supplemented rats.

## Introduction

Many cardiovascular ischaemia/reperfusion injury studies use healthy rats for their research protocols. In clinical conditions, unhealthy diets and lifestyle are normally associated with increased risk of myocardial infarction [1]. Previous studies using healthy animals contributed to elucidating certain mechanisms of cardiovascular protection. However, it has recently been shown that cholesterol feeding reverses the beneficial effects of preconditioning [2]. One such a model is a mildly dislipidaemic rat model, where the rat is supplemented with low doses of cholesterol for a short time period. In this model, peroxynitrite was increased with a subsequent reduction of myocardial function [3]. This ultimately led to contractile failure of the myocardium, or myocardial stunning injury. Additionally, this model was shown to inhibit the cardioprotective effects of myocardial preconditioning [2]. Osipov and co-workers (2009) demonstrated in an *in vivo *hypercholesterolaemic pig model increased infarct size when compared with normal control animals [4]. The authors concluded that increased infarct size in hypercholesterolaemic hearts was associated with increased oxidative stress and inflammation, together with downregulation of cell survival pathways and induction of apoptosis.

Red palm oil (RPO) is an antioxidant rich oil which contains approximately 50% saturated and 50% unsaturated fatty acids [5,6]. Carotenoids and vitamin E (75% of which is tocotrienol) are the most abundant antioxidants in this oil. Both of these antioxidants are contained at a level of at least 500 ppm in RPO [5,6]. The cocktail of antioxidants within RPO is believed to have synergistic effects [7,8]. The oil offers cardioprotection, by activation of several different protective pathways which work synergistically together [9].

Dietary RPO supplementation has previously been shown to offer protection against ischaemia/reperfusion injury in the isolated perfused heart [10-12]. Esterhuyse and co-workers (2005) [10] showed that dietary RPO supplementation could improve post ischaemic functional recovery in rats fed a standard rat chow diet (SRC), and rats fed a SRC plus 2% cholesterol for six weeks [10]. They suggested that RPO mediated protection against ischaemia/reperfusion injury may be induced through different pathways in hearts of SRC fed and cholesterol fed rats.

Matrix metalloproteinases (MMPs) are calcium and zinc dependent endopeptidases. Under normal physiological conditions they facilitate cell migration and tissue remodeling [13]. Recently it was found that MMP2 plays a role in ischaemia/reperfusion damage in the heart [14]. Increased MMP2 activity has also been associated with hypercholesterolaemic diets [2]. This may be due to the increased peroxynitrite production within the myocardium [3]. ROS, and peroxynitrite have been shown to activate MMP2 through redox modification of the regulatory site of this enzyme [15-18]. This redox modification leads to activity being displayed in the 75 and 72 kDa isoforms of the enzyme, while only the 64 kDa isoform is active if MMP2 is activated in the classical manner [16,18]. Activated MMP2 damages cardiomyocytes during reperfusion. This is achieved by cleaving the contractile protein regulatory element, troponin I and possibly other structural and cytoskeletal proteins [19-24]. Activation of MMP2 during an ischaemic insult is therefore normally associated with decreased functional recovery and larger infarct size of the heart [14,25-28]. This has been confirmed through inhibition of MMP2 by antibodies or chemical agents [29-33].

In a recent study [34] it was shown that red palm oil reduced infarct size in a model of ischaemia/reperfusion injury. However, this model investigated only healthy hearts. The intention of the current study was therefore to use a high cholesterol feeding model to ensure that the model is more clinically relevant and to establish confirmation that red palm oil protection are indeed applicable in unhealthy diets, as was previously argued. In all red palm oil fed studies up to date [9-12], results have indicated post-ischaemic involvement of certain cellular biochemical pathways. However, none of these studies provided any evidence of pre-ischaemic protection by red palm oil.

The aims of this study were: 1) to investigate the effects of dietary RPO supplementation on myocardial infarct size in the hearts of rats on a cholesterol-enriched diet and 2) to determine whether MMP2 activity was altered by RPO supplementation, both pre- and post-ischaemically.

## Materials and Methods

All rats received humane animal care in accordance with the Guide for the Care and Use of Laboratory Animals, published by the United States National Institutes of Health (NIH publication 8523, revised 1985).

### Experimental design

Male Wistar rats were divided into four groups. Rats in these groups were placed on the following diets, respectively:

Group 1: Standard rat chow diet for 9 weeks (Norm)

Group 2: 2% cholesterol-enriched diet for 9 weeks (Chol)

Group 3: Standard rat chow supplemented with 200 μl RPO (Norm+RPO) per day for the last 5 weeks of the 9 week period

Group 4: 2% cholesterol-enriched diet for nine weeks supplemented with 200 μl RPO per day for the last 5 weeks of the 9 week period (Chol+RPO)

Rats were individually housed to ensure that each animal received equal amounts of supplements. RPO and supplements were prepared on a daily basis in order to prevent spoiling. Rats were allowed *ad libitum *access to food and water.

### Isolated heart perfusion

After the feeding period, rats were anaesthetized using diethyl ether. Hearts were isolated, mounted on a Langendorff perfusion apparatus, and were perfused at 37°C using a Krebs-Henseleit buffer solution which was constantly gassed with 5% carbon dioxide, 95% oxygen and a constant perfusion pressure of 100 cmH_2_0 was maintained. After mounting, hearts were subjected to 10 minutes of stabilization, followed by 30 minutes of normothermic global ischaemia and 120 minutes of reperfusion. At the end of the perfusion protocol ventricular tissue was frozen at -20°C overnight.

### Infarct size determination

Frozen hearts were cut into 2 mm thick cross-sectional slices. These slices were stained in 2,3,5-triphenyltetrazolium chloride (TTC) for 10 minutes at 37°C. After TTC staining, the slices were transferred to a formalin solution for ten minutes and then placed in phosphate buffer (pH 7.4) [35,36]. Heart slices were then placed between two sheets of glass and scanned into a computer and analyzed using infarct size planimetry software (Infarctsize™ 1.0 Pharmahungary, Szeged, Hungary) in a blinded manner. Infarct size was represented as percentage of the area at risk.

### MMP2 zymography

Coronary effluent collected for 10 minutes before ischaemia and the first 10 minutes of reperfusion was concentrated by ultra filtration using Amicon ultra filtration tubes. The concentrated coronary flow was then subjected to gelatin zymography.

Gelatinolytic activities of MMPs were examined as previously described [18]. Briefly, polyacrylamide gels were copolymerized with gelatin, and a constant amount of protein was separated by electrophoresis in each lane. Following electrophoresis, gels were washed with 2.5% Triton X-100 and incubated for 20 hours at 37°C in incubation buffer. Gels were then stained with 0.05% Coomassie Brilliant Blue in a mixture of methanol/acetic acid/water and destained in aqueous 4% methanol/8% acetic acid. Zymograms were digitally scanned, and band intensities were quantified using Quantity One software (Bio-Rad, Hercules, CA) and expressed as a ratio to the internal standard.

### Serum cholesterol and triglyceride measurement

Serum cholesterol and triglyceride were measured using a test kit supplied by Diagnosticum Zrt. (Budapest, Hungary) as described previously [37].

### Statistics

All values are presented as mean ± SEM. Differences among means were analyzed by two-way ANOVA followed by an appropriate post hoc test to make all pair-wise comparisons and comparisons of each group to the respective control. *P *was considered significant if it was less than 0.05.

## Results

### Animal mass

The body and heart weight of the RPO supplemented rats were significantly decreased when compared to the Norm group and also to the Chol+RPO group after the feeding period (Table 1).

**Table 1:** Animal mass and heart mass after a nine week diet

	Norm	Chol	Norm+RPO	Chol+RPO
**Animal mass (g)**	494.0 ± 18.1	472.6 ± 13.5	381.3 ± 7.6*	511.5 ± 14.4
**Heart mass (g)**	1.6 ± 0.1	1.7 ± 0.1	1.4 ± 0.1*	1.7 ± 0.1

Values are mean ± SEM, n = 8; *p < 0.05 *versus *Norm and Chol+RPO

### Perfusion data

There were no significant differences in the coronary effluent of any of the groups after the supplementation period. Coronary effluent of all groups except the RPO group was significantly decreased after ischaemia, when compared to their baseline values. There were no significant differences in the heart rates before or after ischaemia (Table 2).

**Table 2:** Coronary effluent (CE) collected for 10 min to measure MMP activity and heart rate (HR) before and after ischaemia

	CE before ischaemia (mL/10 min)	CE after ischaemia (mL/10 min)	HR before ischaemia (BPM)	HR after ischaemia (BPM)
**Norm**	205.0 ± 5.7	59.2 ± 4.0*	387.4 ± 56.5	338.0 ± 128.6
**Chol**	185.9 ± 21.7	67.1 ± 6.5*	390.8 ± 37.3	346.0 ± 115.3
**Norm+RPO**	152.5 ± 12.9	100.0 ± 6.9	344.1 ± 12.0	328.0 ± 40.7
**Chol+RPO**	141.9 ± 12.5	61.9 ± 3.8*	327.3 ± 17.6	339.0 ± 29.2

Values are mean ± SEM, n = 8; *p < 0.05 *versus *the corresponding "before ischaemia" values.

### Infarct size

**Figure 1:**
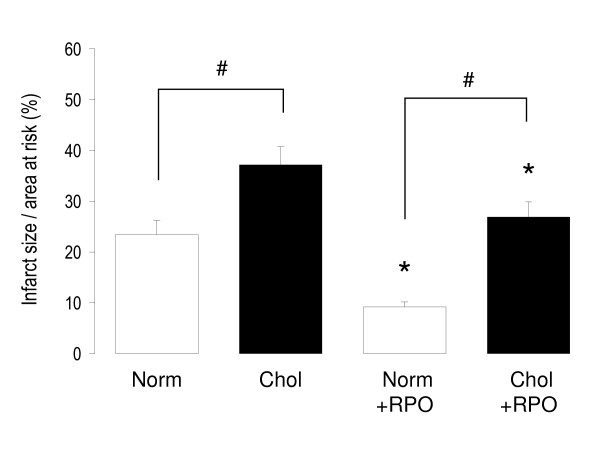
**Myocardial infarct size in rats fed different diets for nine weeks**. Infarct size is expressed as a percentage of the area at risk, values are mean ± SEM, n = 8; *p < 0.05 *versus *corresponding non-RPO treated groups; #p < 0.05 *versus *corresponding normal diet.

Cholesterol-enriched diet alone increased myocardial infarct size from 23.5 ± 3.0% to 37.2 ± 3.6% (p < 0.05) when compared to normal diet. RPO supplementation significantly reduced infarct size in Norm+RPO and also in Chol+RPO (9.2 ± 1.0% and 26.9 ± 3.0%), respectively (Figure 1). Infarct size in Chol+RPO group was comparable to the group fed with normal diet.

### MMP2

**Figure 2:**
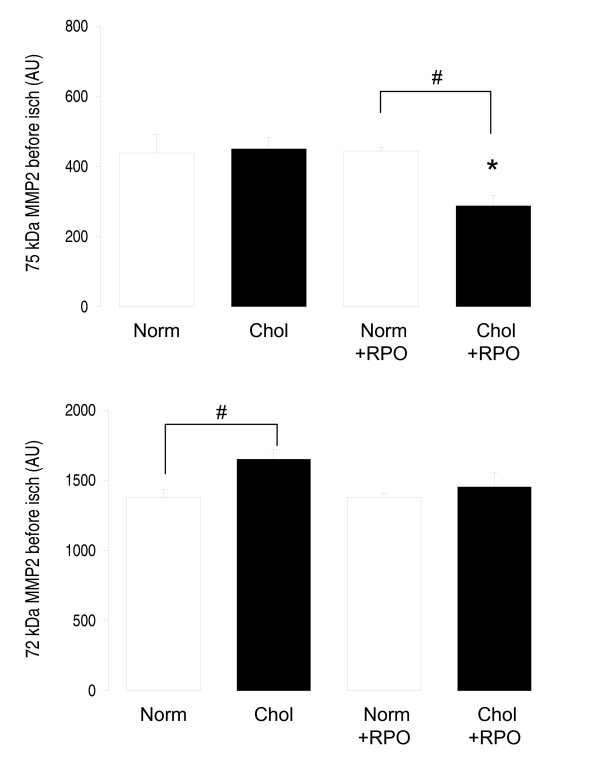
**MMP2 activity in coronary effluent collected for 10 minutes before ischaemia**. Values are mean ± SEM, n = 8; *p < 0.05 *versus *corresponding non-RPO treated groups; #p < 0.05 *versus *corresponding normal diet.

**Figure 3:**
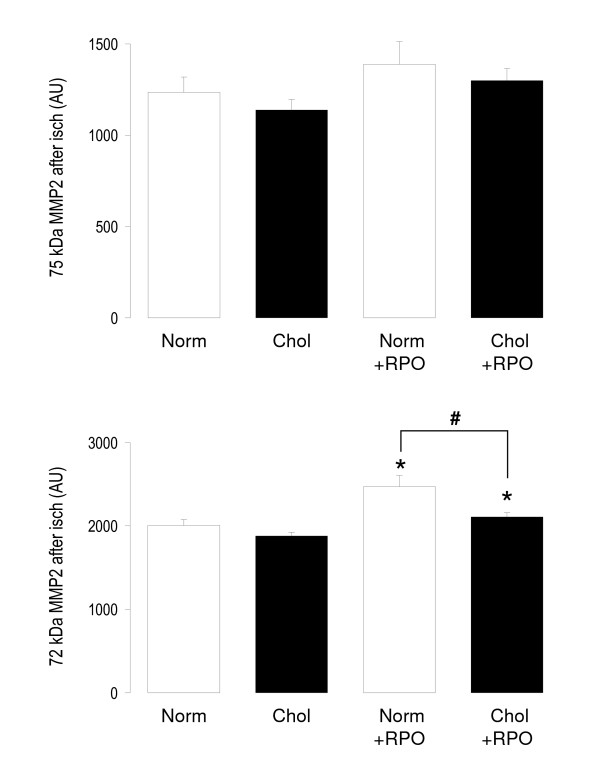
**MMP2 activity in coronary effluent collected for the first 10 minutes of reperfusion**. Values are mean ± SEM, n = 8; *p < 0.05 *versus *corresponding non-RPO treated groups; #p < 0.05 *versus *corresponding normal diet.

Before ischaemia activity of the 75kDa isoform of MMP2 was significantly lower in the Chol+RPO group when compared to the Chol group (228 ± 28 arbitrary units *versus *450 ± 34 arbitrary units; Figure 2). After ischaemia MMP2 (72kDa isoform) activity of the RPO supplemented group was significantly increased when compared to rats with normal diet (2472 ± 132 arbitrary units *versus *2007 ± 68 arbitrary units; Figure 3).

### Serum lipid profile

There were no significant differences in the serum total cholesterol and triglyceride level among groups (Table 3).

**Table 3:** Serum total cholesterol and serum triglyceride levels of rats after nine weeks of diet

	Norm	Chol	Norm+RPO	Chol+RPO
**Total cholesterol** **(mmol/L)**	1.96 ± 0.05	1.91 ± 0.05	2.15 ± 0.23	1.79 ± 0.04
**Triglycerides** **(mmol/L)**	0.72 ± 0.06	0.80 ± 0.08	0.71 ± 0.06	0.80 ± 0.07

Values are mean ± SEM, n = 8.

## Discussion

Our results show that dietary RPO supplementation reversed the negative effects of cholesterol supplementation in the ischaemia/reperfusion rat heart model. Furthermore, dietary RPO supplementation reduced myocardial infarct size in cholesterol supplemented rats. Previous studies have shown that dietary RPO supplementation improved functional recovery of cholesterol fed rats after ischaemia [10,12]. In the present study cholesterol supplementation was carried out for a longer period (nine weeks *versus *six weeks in previous studies). This indicates that RPO could effectively protect hearts against ischaemia/reperfusion injury, despite a longer duration of cholesterol feeding. Osipov and co-workers (2009) found that hypercholesterolaemic pigs had increased left ventricular function throughout the ischaemia/reperfusion period when compared to normal pigs [4]. This was, however, associated with an increased infarct size and increased apoptotic markers. Our results together with previous studies [10,12] showed that dietary RPO can attenuate the harmful effects of cholesterol supplementation in the ischaemia/reperfusion model. Our total serum cholesterol results were not increased in the cholesterol fed rats. This was expected, as a previous study employed a similar model of cholesterol feeding in rats, without achieving significant changes in serum cholesterol [38]. The effects of the cholesterol feeding on the cardiovascular system are however clearly discussed in the study of Giricz and co-workers (2003) through the depletion of nitric oxide, and our current study by increased myocardial infarct size [38].

Coronary flow in the Norm group was reduced by 70%, in the Chol group by 64%, in the Chol+RPO group by 57% and in the Norm+RPO group by 33% after ischaemia. This indicates that RPO attenuated the fall in coronary flow after ischaemia. This may suggest that RPO supplementation improves vascular function during reperfusion.

MMP2 activity was measured before ischaemia in the cholesterol supplemented groups and compared to a Norm control group and a RPO supplemented group, as it may be expected that the cholesterol supplemented groups may have increased oxidative stress after supplementation. As increased oxidative stress leads to activation of MMP2 through redox modification of it's regulatory subunit, this would be associated with changes in MMP2 activity before ischaemia which would be expected to be absent in normal rats [2,15-18]. Our results demonstrate for the first time that dietary RPO supplementation may alter myocardial oxidative stress before ischaemia in cholesterol fed rats, as MMP2 activity was reduced before ischaemia. The reduction in MMP2 activity before ischaemia in rats supplemented with both cholesterol and RPO suggests that RPO was able to reduce oxidative stress in these rats. This would most probably be achieved through quenching of ROS, which is generated in greater proportions in cholesterol supplemented rats [3,38]. Increased generation of ROS and oxidative stress would normally be associated with activation of MMP2 [15,17,18]. As increased activity of MMP2 may lead to either cardiac remodeling, or tissue damage [21-24], this reduction in MMP2 activity may play a role in RPO mediated protection against ischaemia/reperfusion injury. However, MMP2 activity of the Norm+RPO was increased during reperfusion, when compared to normal rats without RPO supplementation. This would normally be associated with increased myocardial susceptibility to ischaemia/reperfusion injury [14,25-28]. RPO was able to reduce myocardial infarct size in cholesterol fed rats, despite increased activity of MMP2 in reperfusion found in normal rats. This suggests MMP2 activity may only play a protective role in cholesterol fed rats, and that other protective pathways are responsible for RPO mediated protection in normal rats.

The aim of this study was to investigate whether MMP2 activity was involved in RPO mediated protection of cholesterol fed rat hearts against ischaemia/reperfusion. Our results suggest that MMP2 activity may play a role in RPO mediated protection of the hearts of the cholesterol fed rats, but not the hearts of SRC fed rats. This suggests that more pathways of protection may play a role in this protection. Kruger and co-workers (2007) [12] found that RPO supplementation of cholesterol fed rats led to decreased phosphorylation of pro-apoptotic molecules, p38 and JNK. This coincided with increased phosphorylation of the pro-survival kinase ERK early in reperfusion, which leads to reduced apoptosis. Apoptosis has been shown to play a role in the detrimental effects of hypercholesterolaemia in the heart. Inhibition of apoptosis may therefore explain the protective effects of RPO in this model [12].

## Conclusion

For the first time we showed that dietary RPO supplementation attenuated increased susceptibility of cholesterol fed rat hearts to ischaemia/reperfusion injury as evidenced by reduced infarct size. Myocardial MMP2 activity was reduced in cholesterol and RPO supplemented rat hearts before ischaemia (Figure 2), but not after ischaemia (Figure 3) associated with decreased infarct size. This may suggest a different or additional mechanism of protection.

## Competing interests

The authors declare that they have no competing interests.

## Authors' contributions

GS was involved in all experimental procedures, and played an important role in writing and editing the manuscript. DB was equally involved in experimental work and drafted the manuscript. KK contributed to all experimental work and data processing. She was also involved in the editing of the manuscript. TC was involved in the planning the study, interpretation of results and editing of the manuscript. CC, AE, PF and JvR were involved in planning the study, interpretation of the results and editing the manuscript. All authors read and approved the final manuscript.
